# Scoring functions and enrichment: a case study on Hsp90

**DOI:** 10.1186/1471-2105-8-27

**Published:** 2007-01-26

**Authors:** Chrysi Konstantinou-Kirtay, John BO Mitchell, James A Lumley

**Affiliations:** 1 Unilever Centre for Molecular Science Informatics, Department of Chemistry, University of Cambridge, Lensfield Rd, Cambridge CB2 1EW, UK; 2 Arrow Therapeutics Ltd., Britannia House, 7 Trinity Street, London, SE1 1DA, UK

## Abstract

**Background:**

The need for fast and accurate scoring functions has been driven by the increased use of *in silico *virtual screening twinned with high-throughput screening as a method to rapidly identify potential candidates in the early stages of drug development. We examine the ability of some the most common scoring functions (GOLD, ChemScore, DOCK, PMF, BLEEP and Consensus) to discriminate correctly and efficiently between active and non-active compounds among a library of ~3,600 diverse decoy compounds in a virtual screening experiment against heat shock protein 90 (Hsp90).

**Results:**

Firstly, we investigated two ranking methodologies, GOLD_rank _and BestScore_rank_. GOLD_*rank *_is based on ranks generated using GOLD. The various scoring functions, GOLD, ChemScore, DOCK, PMF, BLEEP and Consensus, are applied to the pose ranked number one by GOLD for that ligand. BestScore_*rank *_uses multiple poses for each ligand and independently chooses the best ranked pose of the ligand according to each different scoring function. Secondly, we considered the effect of introducing the Thr184 hydrogen bond tether to guide the docking process towards a particular solution, and its effect on enrichment. Thirdly, we considered normalisation to account for the known bias of scoring functions to select larger molecules. All the scoring functions gave fairly similar enrichments, with the exception of PMF which was consistently the poorest performer. In most cases, GOLD was marginally the best performing individual function; the Consensus score usually performed similarly to the best single scoring function. Our best results were obtained using the Thr184 tether in combination with the BestScore_rank _protocol and normalisation for molecular weight. For that particular combination, DOCK was the best individual function; DOCK recovered 90% of the actives in the top 10% of the ranked list; Consensus similarly recovered 89% of the actives in its top 10%.

**Conclusion:**

Overall, we demonstrate the validity of virtual screening as a method for identifying new leads from a pool of ligands with similar physicochemical properties and we believe that the outcome of this study provides useful insight into the setting up of a suitable docking and scoring protocol, resulting in enrichment of '*target active*' compounds.

## Background

Recent years have seen the development of *in silico *'virtual screening' of very large libraries of molecules as an integral part of the drug development process. An initial library might contain millions of compounds that are potentially available, either in-house or from vendors' catalogues. Virtual screening has the twin goals of finding molecules with both favourable ADMET properties and suitable bioactivity. The first goal involves searching for molecules with favourable values of relevant properties such as solubility, polarity, logP, possible toxicity, absorption and likely routes of metabolic breakdown, hence guiding the medicinal chemist towards molecules of good bioavailability and low toxicity. While this is a very important aspect of virtual screening, hereafter we shall concentrate on the second goal of finding molecules with suitable bioactivity.

In the favourable case where the three dimensional structure of the target, usually a protein, is known, it is possible to computationally dock thousands of molecules into the active site, looking for those that will have suitable spatial and chemical complementarity and hence bind strongly [1]. The simplest case is rigid body docking, where we take given fixed conformations of the protein and ligand and find where in the protein the ligand will bind, and how it will be oriented, in order to obtain the (spatially and chemically) best fit. Even with the assumption of rigid bodies, the search space is six dimensional.

However, in reality the problem is harder than this. Both protein and ligand are liable to undergo conformational change upon docking. This means that the method must allow conformational variation, ideally in both molecules. The search space acquires a high dimensionality and the flexible docking problem is difficult. In the present work, we carry out semi-flexible docking.

One of the most successful strategies for docking is to use a genetic algorithm, as in the program GOLD [2]. Such an algorithm mirrors Darwinian evolution, representing the solution as a 'chromosome'. Genetic algorithms allow a population of solutions to exist, and in each 'generation' these can evolve by processes such 'breeding' and 'mutation'. Poor solutions are killed off, while good ones leave their offspring in future generations. Such algorithms may typically reach an excellent solution in a few tens of generations.

Scoring functions, either identical to or different from those utilised as measures of fitness within docking programs, are used to assign predicted binding affinities and rank ligands relative to one another, with a view to selecting and testing experimentally a small subset for biological activity. The development of suitable scoring functions for ranking the solutions produced by docking methods, and especially for accurate prediction of protein-ligand binding affinities, remains a considerable challenge. The scoring function must accurately measure both intramolecular conformational strain energy and intermolecular interaction energy. Several contrasting kinds of scoring function have had some success, including some based on molecular mechanics force fields (Coulomb + van der Waals + hydrogen bonding + bond stretching & bending + torsions) [3] and others centred on modelling each of the relevant terms of a 'Master Equation' describing the free energy of interaction. An alternative is provided by knowledge-based scoring functions, such as BLEEP [4] and PMF [5], where the objective is to use the co-ordinates of hundreds of three dimensional protein-ligand complex structures as a knowledge base. Using this knowledge, a putative protein-ligand interaction geometry can be assessed on the basis of how similar its features are to those of the ensemble of known structures. The features used are the distributions of atom-atom distances between protein and ligand in the complex. Commonly observed features, such as donor/acceptor type nitrogen/oxygen distances at typical hydrogen bonding distances around 3Å, score favourably. Less frequently observed interactions, such as close polar/non-polar contacts, score unfavourably. When the contributions are summed over all pairs of atoms in the complex, the resulting score indicates how much the putative structure 'looks like' a real protein-ligand complex.

When the binding affinity of a series of homologous inhibitors into a particular site is known, it is possible to generate '*customised*' scoring functions to fit the data [6]. Ideally, the combination of the search algorithm and the scoring function should result in a single solution close to the experimental ligand position [7]. General-purpose scoring functions, in contrast, are designed to be applicable to a wide variety of protein-ligand complexes, and are therefore parameterised using a diverse set of protein ligand complexes. This work concentrates on five general-purpose scoring functions.

The application of virtual screening techniques in parallel with High-Throughput Screening (HTS) technology, coupled with structural biology [8], can extend the scope of screening to external databases. This allows more diverse chemical entities to be identified as hits, and as a consequence can help to reduce the assay-to-lead attrition rate observed from HTS [9].

There are many questions, however, associated with the tools employed for docking-based virtual screening. A number of approximations are often employed for the docking/scoring searches (*e.g., *neglect of protein flexibility in rigid docking, lack of a rigorous treatment of solvation, and the choice of one particular protonation state) in order for the virtual screen to be completed within an acceptable time limit, as well as other unavoidable approximations such as the limitations of X-ray crystal structures. Despite the above, virtual screening can be improved by taking into consideration additional information about the receptor of interest and using this information advantageously in docking/scoring applications [10-13]. Recent advances in virtual screening include various physics-based methods [14-16] and consensus scoring [17,18].

In our study, we concentrate on heat shock protein 90 (Hsp90), which is a chaperone and a target for anti-cancer therapeutics [19]. Prior to screening, the binding site was prepared by using the SYBYL^® ^7.0 software of Tripos [20]. The docking program GOLD 2.2 [2] was used to perform docking with and without the presence of a tether. The ligands docked were taken from '*active*' and '*inactive*' datasets [21]; we also used a set of '*decoys*' retrieved from the CIPSLINE cancer database [22]. Post-dock scoring was calculated using multiple scoring functions: GOLD [2], ChemScore [23], DOCK [24], PMF [5], BLEEP [4,25-28], and a Consensus generated from the preceding five. We used two ranking methodologies: best GOLD_*rank *_and BestScore_*rank *_(see Methods).

## Results and discussion

**Figure 1:**
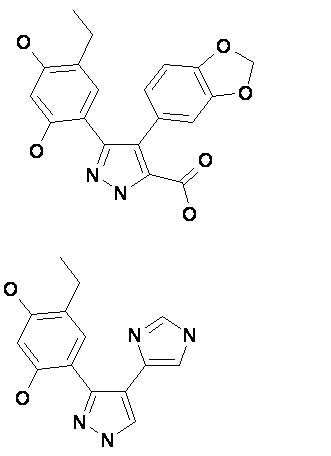
Structures of 4BC (upper) and 43P (lower).

**Figure 2:**
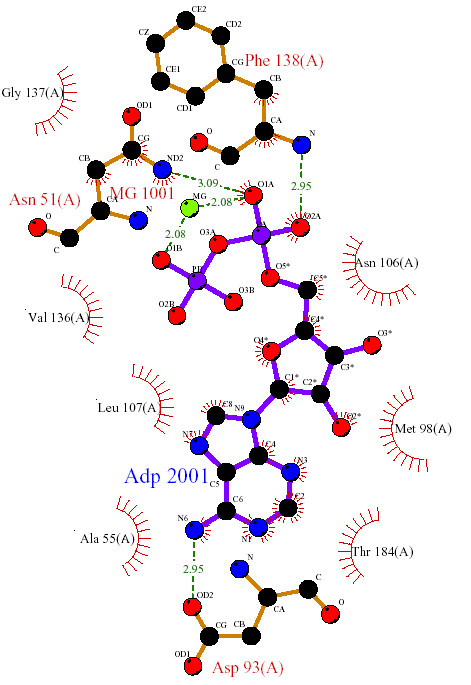
LIGPLOT [38] diagram of the protein-ligand interactions in the Hsp90 N-terminal domain bound with ADP (PDB code: 1BYQ).

We analysed crystal structures (PDB Codes: 1YC1/1YC3/1YC4[29], 1BYQ[30]) containing the ligands 4BC (Figure 1), 43P (Figure 1), and ADP (Figure 2) bound to the N-terminal ATP binding domain of human Hsp90α, as described in detail in the Methods section.

We considered the conformation of the ADP bound Hsp90 (PDB Code: 1BYQ), representing a ligand bound structure, as a suitable starting point for virtual screening. Thr184 was used as a tether, since it generated a low RMSD (root mean square deviation) from its corresponding crystallographic conformations (< 1Å, Table 1) and, in preliminary work, strongly outperformed the alternative *Asp93 *tether. Each library compound was docked to the binding site (PDB Code: 1BYQ), firstly with no tether and secondly with the tether Thr184. We separately used the GOLD and ChemScore functions for on-dock scoring. The RMSD values between the docked ligands and their crystallographic conformations (1YC1/1YC3/1YC4) are given in Table 1.

**Table 1:** Deviations between docked and crystallographic conformations

***Compounds***	***GOLD***	***ChemScore***
***4BC (no tether)***	*1.84 *[1YC1]*/0.38 *[1YC3]	*1.19 *[1YC1]/*1.58 *[1YC3]
***4BC (Thrl84)***	*1. 62 *[1YC1]*/0.51 *[1YC3]	*0.71 *[1YC1]*/0.98 *[1YC3]
***43P (no tether)***	*0.51 *[1YC4]	*0.64 *[1YC4]
***43P (Thrl84)***	*0.78 *[1YC4]	*1.19 *[1YC4]

RMS deviations in Å between the docked conformations of '*active*' compounds 4BC and 43P and their corresponding crystallographic conformations (PDB codes: 1YC1, 1YC3, 1YC4).

A larger set of '*active*' (261) and '*inactive*' (54) compounds similar to 4BC and 43P and a '*decoy*' (~3600) set of known drugs, with similarly druglike physicochemical properties (molecular weight, logP, numbers of hydrogen bond donors, hydrogen bond acceptors and rotatable bonds, see Methods), but assumed inactive against this target, were docked to the binding site, with and without the Thr184 hydrogen bond tether, using GOLD 2.2 and the GOLD scoring function. Post-dock scoring used the SYBYL^®^-CScore™ module, BLEEP as stand alone software, and Consensus (see Methods).

By ranking all ligands *via *their score values, the enrichments were calculated for each scoring function to establish how many decoys had to be picked in order to find all the original actives, based on poses chosen with either the GOLD_*rank *_or BestScore_*rank *_methodologies. GOLD_*rank *_is based on ranks generated using GOLD. Each scoring function is applied to the pose ranked number one by GOLD for that ligand. BestScore_*rank *_chooses, in each case, the best pose as ranked by the particular scoring function in question, rather than always using the pose ranked first by GOLD. The virtual screening was carried out both with and without the tether.

Scores normalised for molecular weight [31] were obtained by dividing the raw score by the number of heavy atoms to the power of 1/3. This is designed to reduce the inherent bias towards larger molecules that arises from the additive nature of scoring functions. Normalisation also reduces the prevalence of high molecular weight molecules amongst the hits, which is likely to be beneficial from a lead optimisation perspective.

**Figure 3:**
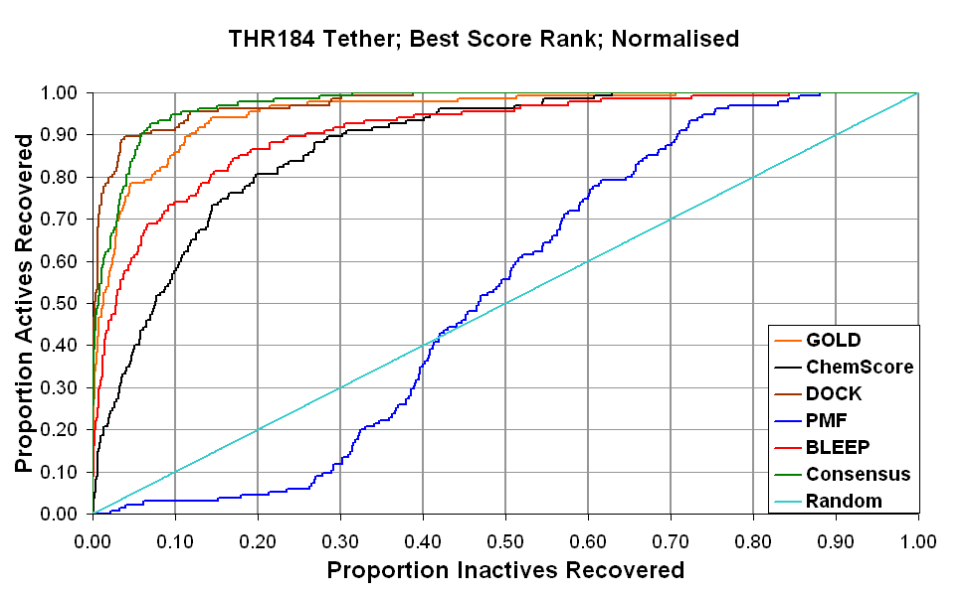
Receiver Operating Characteristic (ROC) curves for the combination of the Thrl84 tether, the BestScore_rank _protocol, and normalisation by dividing the raw score by the number of heavy atoms to the power of 1/3.

We give the results in Table 2 (no tether) and Table 3 (Thr184 tether). Some of the same data are shown as Receiver Operating Characteristic (ROC) curves in Figure 3. The ROC curves are presented as plots of the proportion of all actives recovered versus the proportion of all inactives recovered as one proceeds from the top to the bottom of the ranked list. The areas under these ROC curves are a convenient measure of performance, and are included in Tables 2 and 3. An ideal case would recover all actives before recovering any inactives and hence have an area of unity. The apparent contradiction between the retrievals of actives for GOLD between Tables 2, 3 (*e.g., *28% of actives for GOLD_*rank *_and 25% of actives for BestScore_*rank *_in the top 10% in Table 2) is due to the different implementations of the GOLD algorithm in GOLD 2.2 and in the SYBYL^® ^-CScore^™ ^module.

**Table 2:** Receiver operating characteristic data obtained with no tether

**Scoring Functions**	**GOLD**_*rank*_					**BestScore**_*rank*_				
	**% Actives**					**% Actives**				

	**10**	**20**	**30**	**50**	**AUC**	**10**	**20**	**30**	**50**	**AUC**

***GOLD***	28	42	55	75	.705	25	41	57	76	.715
***ChemScore***	19	39	53	79	.704	21	35	58	84	.725
***DOCK***	23	37	50	73	.678	18	32	44	70	.654
***PMF***	1	8	16	41	.419	0	0	5	27	.364
***BLEEP***	19	35	50	69	.633	17	33	48	76	.666
***Consensus***	24	37	52	72	.681	21	33	51	74	.677
***GOLD ****(normalised)*	27	39	51	71	.674	25	41	54	74	.695
***ChemScore ****(normalised)*	15	29	44	67	.635	13	30	48	77	.677
***DOCK ****(normalised)*	27	41	53	77	.710	20	33	45	70	.653
***PMF ****(normalised)*	0	4	11	37	.385	0	0	4	21	.334
***BLEEP ****(normalised)*	17	29	45	64	.610	16	29	46	71	.648
***Consensus ****(normalised)*	28	43	59	78	.732	23	36	49	75	.682

Retrieval of actives without using a tether. Percentages of actives corresponding to the top 10%, 20%, 30% & 50% of the screened library. The normalised scores were obtained by dividing by the number of heavy atoms to the power of 1/3. AUC is the area under the ROC curve.

**Table 3:** Receiver operating characteristic data obtained with the Thr184 tether

**Scoring Functions**	**GOLD**_*rank*_					**BestScore**_*rank*_				
	**% Actives**					**% Actives**				

	**10**	**20**	**30**	**50**	**AUC**	**10**	**20**	**30**	**50**	**AUC**

***GOLD***	37	61	78	92	.824	63	87	96	99	.919
***ChemScore***	33	52	70	90	.797	56	81	93	99	.898
***DOCK***	32	47	62	81	.753	59	84	92	100	.899
***PMF***	0	6	19	43	.466	3	4	9	53	.505
***BLEEP***	28	44	57	79	.718	45	73	87	98	.867
***Consensus***	39	53	69	92	.803	63	92	97	100	.930
***GOLD ****(normalised)*	43	60	73	88	.806	79	94	98	99	.954
***ChemScore ****(normalised)*	24	44	62	86	.751	52	76	88	96	.878
***DOCK ****(normalised)*	35	49	63	86	.769	90	96	97	100	.976
***PMF ****(normalised)*	0	2	14	41	.448	3	4	13	56	.521
***BLEEP ****(normalised)*	25	39	52	76	.699	69	83	90	96	.909
***Consensus ****(normalised) (normalised)*	38	57	71	89	.801	89	97	99	100	.974

Retrieval of actives with the Thr184 tether. Percentages of actives corresponding to the top 10%, 20%, 30% and 50% of the screened library. The normalised scores were obtained by dividing by the number of heavy atoms to the power of 1/3.

Some clear trends are apparent from these results, based on analysis of the data in Tables 2 and 3, and especially the areas under the relevant ROC curves.

(1) The relative performance of the scoring functions is typically given by

GOLD ≈ Consensus > DOCK ≈ ChemScore > BLEEP >> PMF.

The Consensus score used in this work is a simple sum of the Z-scaled scores from five scoring functions. As such, it is less sophisticated than other consensus strategies considered elsewhere [17,27]. Nonetheless, it is generally a robust method, comparable in performance to the best individual scoring function. The good performance of the Consensus scoring method result is to some extent in agreement with recent virtual screening studies where Consensus scoring improves the enrichment of true hits [32-34] in various systems. However, the improvement given by using the Consensus method is small, and on occasions Consensus fails to outperform the best individual function.

The performance of PMF here is usually worse than random and PMF is consistently the poorest performer in all applied protocols. We used the implementation of PMF in SYBYL^® ^7.0; our previous use of the SYBYL implementation of PMF also gave disappointing results [27], though the present ones are certainly poorer. PMF gave much better results in its authors' own in-house implementation [5,35].

(2) The tethered results are in all cases better than the corresponding untethered ones. This effect is particularly strong when the BestScore_*rank *_protocol is used.

The utilisation of tethering during docking requires prior knowledge of ligand-protein X-ray structures, which is not always available. Inspection of the structures shows that in general the tether is satisfied as expected in the better scoring structures and structures unable to satisfy the tether appear further down the ranked list.

(3) When the tether is used, the BestScore_*rank *_protocol always gives better results than a corresponding calculation using the GOLD_*rank *_protocol. For untethered docking, there is little difference in the performance of the two protocols.

The GOLD_*rank *_protocol tends to be biased towards to the GOLD function in relation to the other scoring functions; BestScore_*rank *_proved to be an unbiased method selecting the best score for each scoring function independently.

(4) In most cases, normalisation has little effect on performance, and any such effect is often deleterious. However, for the particular combination of tethered docking and the BestScore_*rank *_protocol, normalisation gives a significant improvement (though not for ChemScore).

(5) This combination of tethered docking, the BestScore_*rank *_protocol and normalisation by dividing the raw score by the number of heavy atoms to the power of 1/3 gives the best results found in this study; this is true for every scoring function except ChemScore.

We consider that this optimal combination gives a good virtual screening performance (other than with PMF), with the percentages of actives found in the first 10% of the ranked library being 90%, 79%, 69% and 52% for the four best individual scoring functions and 89% for Consensus. The ROC curves for this combination are shown in Figure 3.

(6) The performance ranking of the scoring functions for this optimal combination of tether, BestScore_*rank *_and normalisation is somewhat atypical of those found in our other calculations and is given by

DOCK ≈ Consensus > GOLD > BLEEP > ChemScore >> PMF.

## Conclusion

This work has demonstrated the successful development of a virtual screening methodology, as has been achieved by other groups for different therapeutically relevant targets [36,37]. A library of ~3600 compounds was docked semi-flexibly into the active site of Hsp90. Five scoring functions, including BLEEP, were used to discriminate active from inactive compounds. The present work offers alternative protocols for virtual screening of chemical libraries with an emphasis on the effect of using multiple ligand poses for scoring with some of the most common scoring functions and also tethered and un-tethered docking.

For tethered docking, we find that consideration of multiple poses for each ligand in our BestScore_*rank *_protocol is superior to relying on the best scoring pose generated by a single scoring function. The different scoring functions are thus judged on the basis of their own top-scoring poses, which may be different from one another.

Though normalisation has little effect on enrichment elsewhere in this work, in the case where the Thr184 tether is combined with the BestScore_rank _protocol, normalisation generates a significant improvement in enrichment. This combination of tethered docking, the BestScore_*rank *_protocol and normalisation gives the best results found in this work. Normalisation also reduces the prevalence of high molecular weight molecules amongst the hits, which is likely to be beneficial from a lead optimisation perspective.

Although we use only a very simple implementation of Consensus scoring, we find it to be a generally robust methodology. It performs similarly to the best individual scoring function in each virtual screening run.

Overall, we demonstrate the validity of virtual screening as a method for identifying new leads from a pool of ligands with similar physicochemical properties and we believe that the outcome of this study provides useful insight into the setting up of a suitable docking/scoring protocol, resulting in enrichment of '*target active*' compounds.

## Methods

### Data preparation

Prior to docking-based virtual screening, the binding site was prepared using SYBYL^® ^7.0. Protonation states as at pH7, atom- and bond-types, hydrogen addition, and consideration of active site waters for inclusion/exclusion were implemented using SYBYL^® ^7.0 for the crystal structures (PDB Codes: 1YC1/1YC3/1YC4 [29], 1BYQ[30]) containing the ligands 4BC (Figure 1), 43P (Figure 1), and ADP bound to the N-terminal ATP binding domain of human Hsp90α. A diagram featuring the key protein-ligand interactions for the ligand ADP was generated using LIGPLOT [38] (Figure 2).

For the purpose of this study, we considered the conformation of the ADP bound Hsp90 (PDB Code: 1BYQ), representing a ligand bound structure and a more suitable starting point for virtual screening. The bound inhibitor was not included in the docking run. The 3D coordinates of the ligands were generated from 2D structures using CORINA [39]. For tether selection, we considered Thr184 [29,40] Asp93 [41] and Gly97, which all form distinct hydrogen bonds with bound ligands. Gly97 was not included in the final calculations as it did not form any interaction with ATP and showed poor results in initial validation studies. Default tether weights were used. Both Thr184 and Asp93 initially seemed to be appropriate tethers, since they generated the lowest RMSD from their corresponding crystallographic conformation (< 1Å) for this system (see Table 1). However, our early results showed that the Asp93 tether did not produce a significant enrichment of actives and hence it was not considered further.

### Docking and scoring protocol

Each compound in the library was docked to the binding site (PDB Code: 1BYQ), with no tether and with the Thr184 tether. In each case, we separately used both the GOLD and ChemScore functions for on-dock scoring. The RMSD was calculated between each docked ligand and its later published crystallographic conformation (PDB Codes: 1YC1/1YC3/1YC4) (Table 1 and Figures 3, 4). GOLD_*rank *_is based on ranks generated using GOLD. The various scoring functions, GOLD, ChemScore, DOCK, PMF, BLEEP and Consensus, are applied to the pose ranked number one by GOLD for that ligand. BestScore_*rank *_uses multiple poses for each ligand, and independently chooses the best ranked pose of the ligand according to each individual scoring function.

A larger set of '*active*' (261) and '*inactive*' (54) compounds similar to 4BC (4-benzo[1,3]dioxol-5-yl-5-(5-ethyl-2,4-dihydroxy-phenyl)-2H-pyrazole-3-carboxylic acid) and 43P (4-ethyl-6-[4-(1H-imidazol-4-yl)-1H-pyrazol-3yl]-benzene-1,3-diol) and a '*decoy*' (~3600) set of known drugs (with similar physicochemical properties but assumed inactive against this target) were docked to the binding site, with and without the Thr184 hydrogen bond tether, using GOLD 2.2 and the GOLD scoring function, determined to give the best results in previous test runs. Post-dock scoring using a set of different scoring functions was applied using the SYBYL^®^-CScore™ module, and BLEEP as stand alone software. The Consensus was defined as follows: the mean of each scoring function was subtracted from the score of each compound and divided by its standard deviation; the sum of these scaled values of the five scoring functions generated the consensus. This sum over Z-scaled scoring function values was implemented using perl scripts.

Using the activity data reported in a set of recent patents [42], we defined compounds as 'active' if IC50 < 10 μM and as 'inactive' if IC50 > 50 μM. The 'decoy' set was selected from the CIPSLINE cancer database using the dbtranslate and dbslnfilter tools in SYBYL^® ^7.0, so as to maintain the same physicochemical properties as the active/inactive set: MWt 150–750, logP 1–6, rotatable bonds 0–14 and hydrogen bond donors/acceptors 0–8/0–12. In each case, up to 10 poses were saved for each docked compound. Two separate results files were written containing all poses for all ligands, and containing only the top GOLD_*rank *_pose for each ligand.

## Authors' contributions

CKK carried out the work under the supervision of JAL (industrial supervisor) and JBOM (academic supervisor). All authors cooperated in the analysis of the results and the writing of the manuscript.
